# Prolonged Post-Polymerization Biocompatibility of Polymethylmethacrylate-Tri-n-Butylborane (PMMA-TBB) Bone Cement

**DOI:** 10.3390/ma14051289

**Published:** 2021-03-08

**Authors:** Juri Saruta, Ryotaro Ozawa, Kosuke Hamajima, Makiko Saita, Nobuaki Sato, Manabu Ishijima, Hiroaki Kitajima, Takahiro Ogawa

**Affiliations:** 1 Weintraub Center for Reconstructive Biotechnology, Division of Advanced Prosthodontics, UCLA School of Dentistry, Los Angeles, CA 90095-1668, USA; saruta@kdu.ac.jp (J.S.); ozrt1021@gmail.com (R.O.); hamajima.k0329@gmail.com (K.H.); saita@kdu.ac.jp (M.S.); nobuakisato19@gmail.com (N.S.); manab612@gmail.com (M.I.); hiroaki_k_0315@yahoo.co.jp (H.K.); 2 Department of Oral Science, Graduate School of Dentistry, Kanagawa Dental University, 82 Inaoka, Yokosuka 238-8580, Kanagawa, Japan; 3 Department of Oral Interdisciplinary Medicine (Prosthodontics & Oral Implantology), Graduate School of Dentistry, Kanagawa Dental University, 82 Inaoka, Yokosuka 238-8580, Kanagawa, Japan; 4 Department of Orthodontics, School of Dentistry, Aichi Gakuin University, 1-1-100 Kusumoto-cho, Chikusa-ku, Nagoya 464-8650, Aichi, Japan; 5 Department of Oral Pathology, School of Dentistry, Aichi Gakuin University, 1-1-100 Kusumoto-cho, Chikusa-ku, Nagoya 464-8650, Aichi, Japan; 6 Department of Oral and Maxillofacial Surgery, Graduate School of Medicine, Yokohama City University, 3-9 Fukuura, Kanazawa-ku, Yokohama 236-0004, Kanagawa, Japan

**Keywords:** bone cement, benzoyl peroxide, free radical, polymethylmethacrylate, tri-n-butylborane, orthopedic implants, osteoblast

## Abstract

Polymethylmethacrylate (PMMA)-based acrylic bone cement is commonly used to fix bone and metallic implants in orthopedic procedures. The polymerization initiator tri-n-butylborane (TBB) has been reported to significantly reduce the cytotoxicity of PMMA-based bone cement compared to benzoyl peroxide (BPO). However, it is unknown whether this benefit is temporary or long-lasting, which is important to establish given that bone cement is expected to remain in situ permanently. Here, we compared the biocompatibility of PMMA-TBB and PMMA-BPO bone cements over several days. Rat femur-derived osteoblasts were seeded onto two commercially-available PMMA-BPO bone cements and experimental PMMA-TBB polymerized for one day, three days, or seven days. Significantly more cells attached to PMMA-TBB bone cement during the initial stages of culture than on both PMMA-BPO cements, regardless of the age of the materials. Proliferative activity and differentiation markers including alkaline phosphatase production, calcium deposition, and osteogenic gene expression were consistently and considerably higher in cells grown on PMMA-TBB than on PMMA-BPO, regardless of cement age. Although osteoblastic phenotypes were more favorable on older specimens for all three cement types, biocompatibility increased between three-day-old and seven-day-old PMMA-BPO specimens, and between one-day-old and three-day-old PMMA-TBB specimens. PMMA-BPO materials produced more free radicals than PMMA-TBB regardless of the age of the material. These data suggest that PMMA-TBB maintains superior biocompatibility over PMMA-BPO bone cements over prolonged periods of at least seven days post-polymerization. This superior biocompatibility can be ascribed to both low baseline cytotoxicity and a further rapid reduction in cytotoxicity, representing a new biological advantage of PMMA-TBB as a novel bone cement material.

## 1. Introduction

Our aging society is contributing to a rapid increase in the number of fractures and prevalence of degenerative changes caused by osteoporosis and osteoarthritis [1,2,3,4,5]. As a result, the need for joint replacement/reconstruction is increasing, with over one million shoulder, elbow, hip, and knee replacements performed annually in the United States alone [6,7,8,9]. Polymethylmethacrylate (PMMA)-based acrylic bone cement is used to fix metallic implants or other prostheses in bone during orthopedic procedures [10,11].

PMMA-based bone cement is non-resorbable and mainly composed of PMMA powder and methyl methacrylate (MMA) monomer liquid, together with benzoyl peroxide (BPO) as a polymerization initiator [12]. The powder and liquid components are mixed, and, when doughy, delivered around metallic implants and/or directly injected into a host site to set in situ. Although PMMA-based bone cement is a bio-tolerant material, it has notable side-effects and can result in cellular, tissue, and system-level complications [13,14,15,16,17]. Chemical toxins, including but not limited to the residual monomer after polymerization and the free radicals generated during polymerization, are the main sources of adverse events [18,19,20,21,22,23,24,25]. Adding other constituents, such as growth factors, calcium phosphate, and antioxidants, may improve the overall biological property of PMMA-based bone cement by counteracting the toxicity [20,26,27,28,29]; however, it is far from the solution.

To directly mitigate the toxicity of PMMA-based materials, efforts have been made to improve their inherent biocompatibility. For example, tri-n-butylborane (TBB) has been tested as an alternative polymerization initiator to BPO and has exhibited significantly reduced cytotoxicity, with the number of osteoblasts surviving over the first 24 h of culture significantly higher on PMMA-TBB compared to PMMA-BPO [30]. Furthermore, PMMA-TBB increased the ability of osteoblasts to proliferate and differentiate. However, it remains uncertain whether the advantage of PMMA-TBB is solely due to the immediate interactions between the cells and bone cement, or whether the positive effects persist over time. This is clinically relevant, because ideally the bone cement delivered during surgery should function over long periods of time, or even permanently. Furthermore, addressing this question could help to establish exactly how PMMA-TBB exerts its favorable biological properties. Although long-term studies, such as six-month or five-year follow-ups, are ideal to fully address the question, this study was designed to focus on a short-term exploratory examination after bone cement polymerization. Therefore, in pursuit of development of a new biocompatible bone cement, the objective of this study was to examine and compare the biological properties of an experimental PMMA-TBB bone cement material and two PMMA-BPO bone cements mixed and polymerized over one to seven days.

## 2. Materials and Methods

### 2.1. Bone Cement Preparation

Bone cement specimens were prepared as instructed by the manufacturers and as described previously [30]. The constituents of three different bone cement materials used are shown in Table 1. The materials of PMMA, MMA, and TBB used in this study were manufactured and provided by Mitsui Chemicals, Inc. (Tokyo, Japan). Three bone cement specimens were prepared by mixing and polymerizing in 12-well polystyrene cell culture plates for one, three, and seven days before cell seeding. During polymerization, the bone cement materials in culture plates were kept under dark, ambient conditions at room temperature.

### 2.2. Rat Femur-Derived Osteoblasts Cell Culture

Osteoblasts were obtained from femurs of 10-week-old male Sprague Dawley following the method described elsewhere [31,32,33]. Cells were seeded onto either BPO material or TBB materials placed in 12-well culture dishes at a density of 6 × 10^4^ cells/cm^2^. All experiments were performed following a protocol approved by the UCLA Animal Care and Use Training Manual guidelines (ARC #2005-175-41E, approved on 30 January 2018).

### 2.3. Initial Cell Behavior

Initial cell attachment was measured as the number of cells attached to bone cement surfaces two days after cell seeding. Proliferated cells were also measured as cell density on day five of culture, using a water-soluble tetrazolium salt (WST-1)-based colorimetric assay (WST-1, Roche Applied Science, Mannheim, Germany). The amount of formazan product was measured using an automated microplate reader (Synergy™ HT; BioTek Instruments, Inc., Winooski, VT, USA) at 450 nm.

### 2.4. Morphological Observation of Osteoblasts

The cell behavior of osteoblasts seeded onto bone cement materials were visualized and evaluated using fluorescence microscopy (DMI 4000 B, Leica Microsystems Inc., Buffalo Grove, IL, USA). On day two of culture, the cells were dual stained with fluorescent dyes: 4′,6-diamidino-2-phenylindole (DAPI, Vector Laboratories, Burlingame, CA, USA) for nuclei and rhodamine phalloidin for actin filaments (Molecular Probes, Eugene, OR, USA). The area, perimeter, and Feret’s diameter were measured using an ImageJ (ver.1.51j8, NIH, Bethesda, MD, USA).

### 2.5. Alkaline Phosphatase (ALP) Activity and ALP Staining

The ALP activity of osteoblasts was examined on day five of culture using an ALP stain visual observation and colorimetry-based assay. For visualizing ALP activity, cultured cells were washed and then incubated with Tris buffer containing naphthol AS-MX phosphate and fast red TR salt for 30 min at 37 °C. For colorimetry, the culture was treated with 250 µL p-nitrophenylphosphate (LabAssay ALP, Wako Pure Chemicals, Richmond, VA, USA). The ALP activity was measured at 405 nm using an automated microplate reader (Synergy™ HT, BioTek Instruments, Inc.).

### 2.6. Mineralization Assay

The mineralization capability of osteoblasts was evaluated by visualizing the mineralized area using an Alizarin red staining and colorimetry-based assay on day 15. The bone cement specimens were washed and stained using 1% Alizarin red (pH 6.3–6.4). For colorimetry, cultures were incubated in HCl solution (Sigma Aldrich, St. Louis, MO, USA). The solution was mixed with *o*-cresolphthalein complexone in an alkaline medium (Stanbio LiquidColor, Stanbio, Boerne, TX, USA). Color intensity was measured at 550 nm using an automated microplate reader (Synergy™ HT, BioTek Instruments, Inc.).

The area and intensity of mineralization within osteoblastic cultures were also examined by chemical detection and Ca and P elements. After 15 days of osteoblastic culture on seven-day-old bone cements, the elemental composition of the cultured matrix was analyzed by energy dispersive X-ray spectroscopy (EDS) (ESCA3200; Shimadzu, Tokyo, Japan), as described previously [34].

### 2.7. Bone-Related Genes Expression

Total RNA was extracted and purified from cultures on days 5 and 15, as described previously [35,36]. Real-time polymerase chain reaction (PCR) was performed with the QuantStudio™ 3 Real-Time PCR System (Applied Biosystems, Waltham, MA, USA) to quantify the expression of collagen type I alpha 1 (*Col1a1*; assay ID: Rn01463848_m1, Applied Biosystems), osteocalcin (*Bglap*; assay ID: Rn00566386_g1, Applied Biosystems), Runt-related transcription factor 2 (*Runx2;* assay ID: Rn01512298_m1, Applied Biosystems), and Osterix (*Sp7*; assay ID: Rn02769744_s1, Applied Biosystems) mRNA. Glyceraldehyde-3-phosphate dehydrogenase (*Gapdh*; Assay ID: Rn01775763_g1, Applied Biosystems) was used as the endogenous control.

### 2.8. Polymerization Radical Detection by Electron Spin Resonance Spectroscopy (ESR)

The production of free radicals during polymerization was evaluated using electron spin resonance spectroscopy (ESR). The identification and quantification of the component signals in the spectra were performed based on previously published methods [37,38]. Bone cement samples were investigated using a JES-RE 3X, X-band spectrometer (JEOL, Tokyo, Japan) and a WIN-RAD ESR Analyzer (Radical Research, Tokyo, Japan).

### 2.9. Statistical Analysis

All in vitro culture studies and measurement of free radical levels, except cell morphology, were performed in triplicate with *n* = 3. Cytomorphometry was performed on six cells (*n* = 6). All statistical analyses were performed using SPSS (version 22.0; SPSS Inc., Chicago, IL, USA), and the results are expressed as means ± standard deviations (SD). One-way analysis of variance (ANOVA) was used to assess the differences among three bone cement types. Furthermore, when different time points were involved, two-way analysis of variance (ANOVA) was used to assess the differences among three bone cement types and different time points. When appropriate, Bonferroni’s test was used as a post-hoc test. *p*-values less than 0.05 were considered statistically significant.

## 3. Results

### 3.1. Initial Cell Attachment to Bone Cement

**Figure 1:**
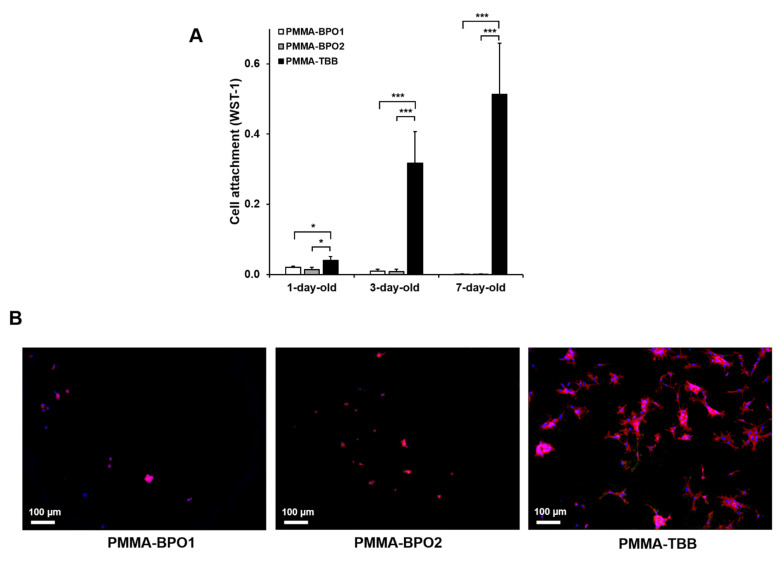
The initial cellular attachment of osteoblasts on the three different types of bone cements (two PMMA-BPO cements and one PMMA-TBB cement) prepared for different amounts of time. Osteoblasts were seeded onto one-day, three-day, and seven-day post-polymerization bone cement specimens. (**A**) The number of cells attached to bone cements on day two of culture evaluated by the water-soluble tetrazolium salt-1 (WST-1) assay. Data are shown as mean ± SD (*n* = 3). Statistical differences between the three groups are shown (two-way ANOVA followed by Bonferroni test, * *p* < 0.05, *** *p* < 0.001). (**B**) Fluorescently stained osteoblasts on day two of culture on seven-day-old bone cement specimens. Representative fluorescence microscopy images of cells visualized with rhodamine phalloidin for actin filaments (red) and 4′,6-diamidino-2-phenylindole (DAPI) for nuclei (blue) are shown. Scale bar = 100 µm. PMMA-BPO, polymethyl methacrylate-benzoyl peroxide; PMMA-TBB, polymethyl methacrylate-tri-n-butylborane.

The number of osteoblasts surviving and attaching to three different bone cements was evaluated using the WST-1 assay after two days of culture (Figure 1A). The number of osteoblasts attaching to PMMA-TBB was significantly greater than those attaching both PMMA-BPO cements, regardless of their age. Of note, there was a significant increase in cell attachment on three-day-old and seven-day-old PMMA-TBB compared to one-day-old PMMA-TBB (*p* < 0.01). Fluorescent microscopy of cells on seven-day-old bone cements confirmed a greater number of cells attached to PMMA-TBB (Figure 1B).

### 3.2. Cell Spreading Behavior

**Figure 2:**
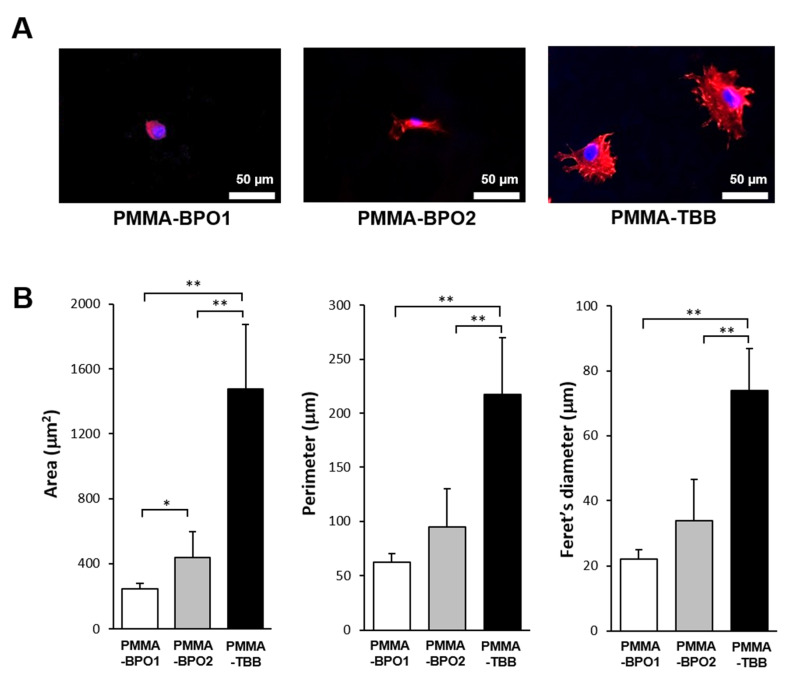
Osteoblastic behavior on bone cement materials. Cytomorphology and cytomorphometry were performed on seven-day-old bone cement specimens. (**A**) Fluorescence microscopy images of osteoblasts on day two of culture following immunochemical staining for cytoskeletal actin (red) and nuclei (blue). Scale bar = 50 µm. (**B**) Histograms for cytomorphometric parameters measured from the images. Each value represents the mean ± SD (*n* = 6). Statistical differences between the three groups are shown (one-way ANOVA followed by Bonferroni test, * *p* < 0.05, ** *p* < 0.01).

The spreading behavior of osteoblasts on seven-day-old bone cements was qualitatively and quantitatively analyzed after two days of culture. Osteoblasts attaching to PMMA-TBB were larger than those attaching to PMMA-BPO cements, with cell membranes projecting in multiple directions indicative of the development of filopodia- and lamellipodia-like structures (Figure 2A). By contrast, cells grown on both PMMA-BPO cements remained circular or oval with no discernible cytoplasmic projections. Cytomorphometry confirmed that the area, perimeter, and Feret’s diameter of cells were greater on PMMA-TBB than on both PMMA-BPO bone cements (Figure 2B).

### 3.3. Cell Proliferation

**Figure 3:**
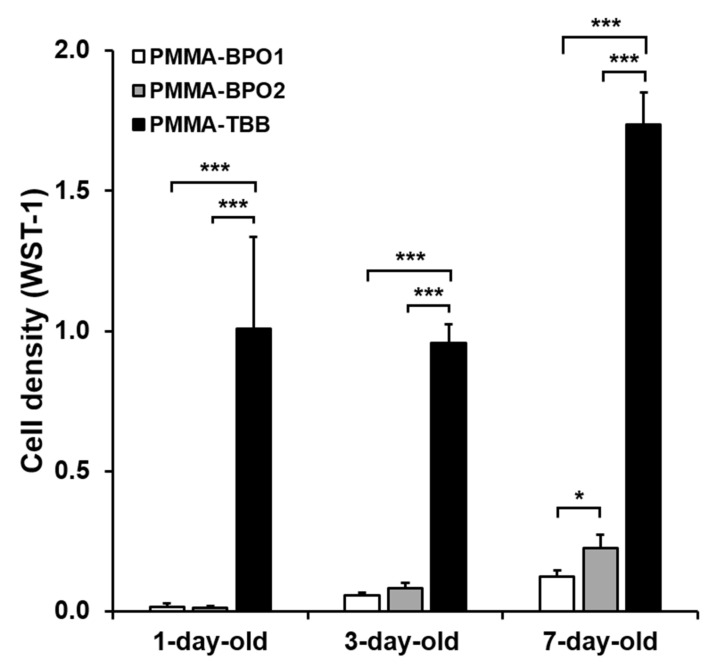
Proliferation activity of osteoblasts on different bone cement types of different ages as evaluated by the WST-1 assay on day five of culture. Data are shown as mean ± SD (*n* = 3). Statistical differences between the three groups are shown (two-way ANOVA followed by Bonferroni test, * *p* < 0.05, *** *p* < 0.001).

Cell proliferation was evaluated by measuring WST-1 values after five days of culture (Figure 3). In contrast to the day two culture values, more cells were present on all three bone cements as they aged. Similar to the day two cell attachment results, cell density was considerably higher on PMMA-TBB than on both PMMA-BPO cements, regardless of age, and even on one-day-old material.

### 3.4. Osteoblastic Differentiation

**Figure 4:**
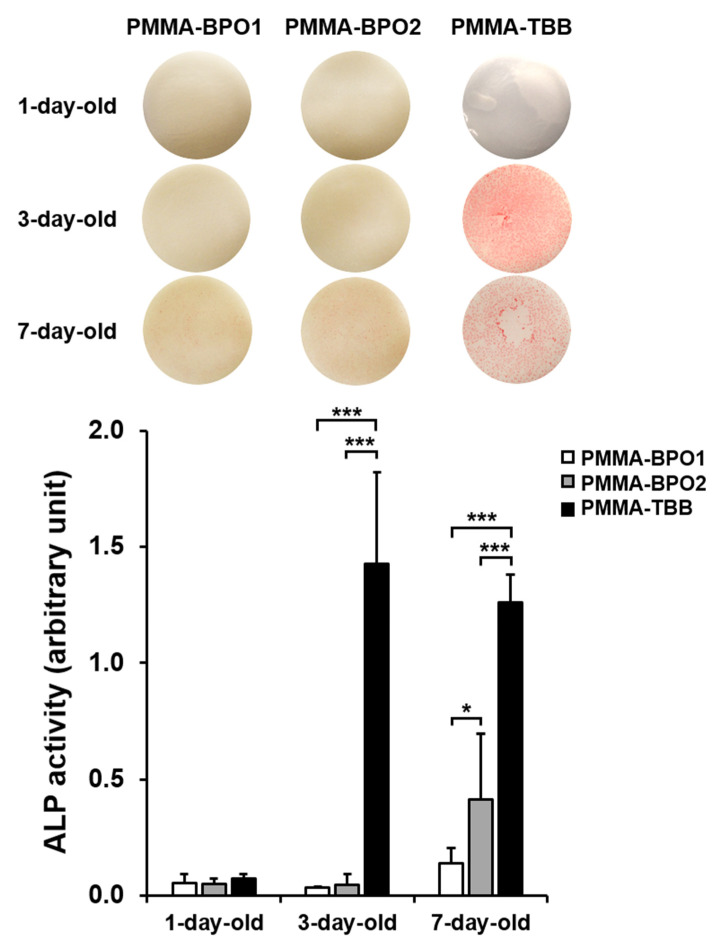
Alkaline phosphatase (ALP) activity of osteoblasts on different bone cement types of different ages evaluated on day five of culture. Representative images of the stained cultures are presented (**top**). ALP activity colorimetrically quantified and standardized relative to cell number is shown in the histogram (**bottom**). Data are shown as mean ± SD (*n* = 3). Statistical differences between the three groups are shown (two-way ANOVA followed by Bonferroni test, * *p* < 0.05, *** *p* < 0.001).

The rate and degree of osteoblastic differentiation were assessed according to three functional phenotypes: alkaline phosphatase (ALP) activity, matrix calcium deposition, and osteogenic gene expression. ALP activity tended to be higher on older materials for all bone cement types (Figure 4). Consistent with the cell attachment and density results, ALP activity was substantially greater from cells cultured on PMMA-TBB than on PMMA-BPO cements, regardless of their age. Of note, the ALP activity increased substantially between one-day-old and three-day-old PMMA-TBB cements (*p* < 0.01), whereas it increased between three-day-old and seven-day-old PMMA-BPO cements (*p* < 0.05). All these quantitative results were supported by the images of ALP staining (Figure 4).

**Figure 5:**
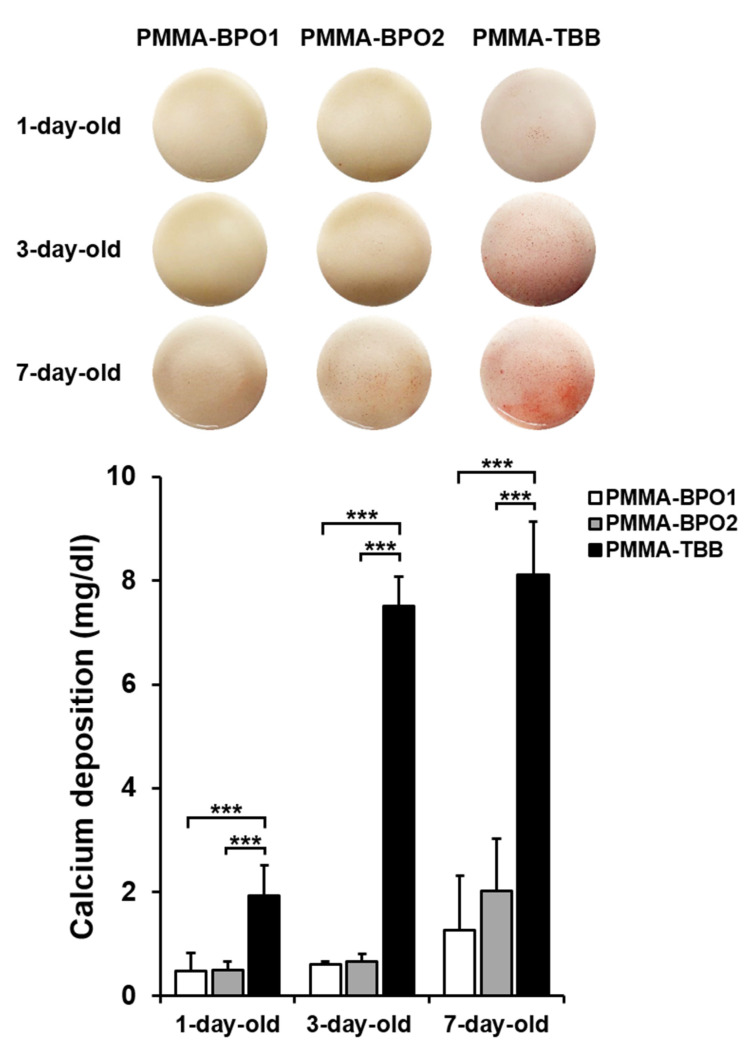
Matrix calcium deposition within osteoblastic cultures. Representative images of Alizarin red-stained cultures on day 15 of culture (**top**) and the result of colorimetric detection of total calcium deposition performed on the same day are presented. Data are shown as mean ± SD (*n* = 3). Statistical differences between the three groups are shown (two-way ANOVA followed by Bonferroni test, *** *p* < 0.001).

Matrix calcium deposition mirrored the ALP activity (Figure 5). Calcium deposition was consistently higher on PMMA-TBB cement than PMMA-BPO cements regardless of age, and calcium deposition on one-day-old PMMA-TBB was equivalent to that on seven-day-old PMMA-BPO cements. Similar to the result of ALP activity, a major increase was found between one-day-old and three-day-old PMMA-TBB cements (*p* < 0.01) and between thee-day-old and seven-day-old PMMA-BPO cements (*p* < 0.05). The colored images of Alizarin red staining re-affirmed the superior calcium deposition on PMMA-TBB cements (Figure 5).

**Figure 6:**
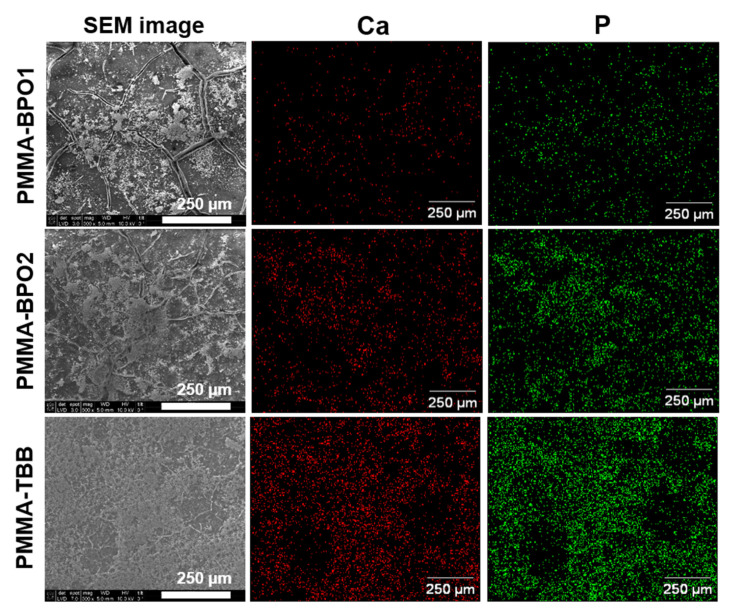
Morphology and chemistry of osteoblastic cultures on bone cements. Osteoblasts were cultured on seven-day-old bone cement specimens. Scanning electron microscopy (SEM) images of cultures on day 15 and the energy-dispersive X-ray spectroscopy (EDX) mapping taken from the corresponding area to detect the atomic Ca and P are presented side-by-side. Scale bar = 250 µm.

The cultured matrix was also examined by SEM and EDX (Figure 6). Biological structures were found extensively on PMMA-TBB cements compared with PMMA-BPO cements in the SEM images. Cultures on PMMA-BPO bone cements tended to exhibit bare bone cement surfaces without biological structures. The biological structures on PMMA-TBB showed intense Ca and P signals, indicative of a mineralized matrix.

**Figure 7:**
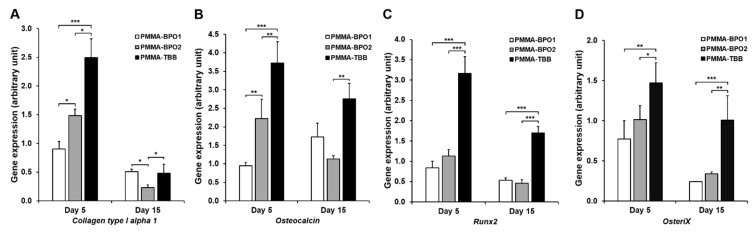
The expression levels of osteoblastic genes, collagen type 1 (**A**), osteocalcin (**B**), runx2 (**C**), and osterix (**D**), quantified by real-time PCR on days 5 and 15 of culture. Osteoblasts were cultured on seven-day-old bone cement specimens. Data are shown as mean ± SD (*n* = 3). Statistical differences between the three groups are shown (one-way ANOVA followed by Bonferroni test, * *p* < 0.05, ** *p* < 0.01, *** *p* < 0.001).

The gene expression of four bone-related genes was measured in cells grown for five and fifteen days on seven-day-old bone cement materials (Figure 7). There was a general trend towards upregulated expression of all genes on PMMA-TBB compared to both PMMA-BPO cements. Specifically, expression of collagen type 1, an early-stage osteoblastic differentiation gene, was upregulated on PMMA-TBB on day five but not on day fifteen; osteocalcin, a late-stage osteoblastic differentiation gene, was upregulated on PMMA-TBB on both days five and fifteen; and *Runx2* and *Osterix*, osteoblastic differentiation transcription factors, were also consistently upregulated at both timepoints on PMMA-TBB.

### 3.5. Free Radical Production by Bone Cements

**Figure 8:**
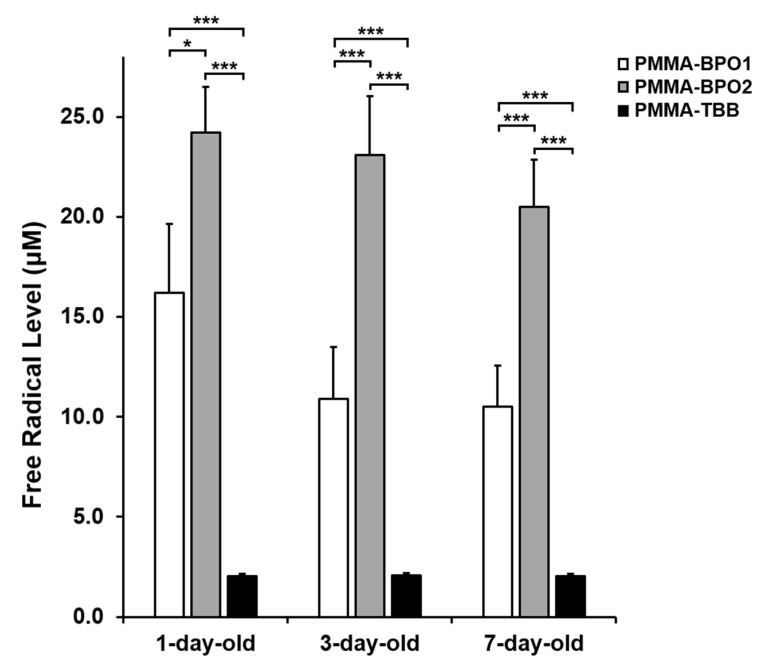
Free radical generation from the three different bone cements prepared for different times evaluated by electron spin resonance spectroscopy (ESR). Data are shown as mean ± SD (*n* = 3). Significant difference shown only compared with PMMA-TBB (two-way ANOVA followed by Bonferroni test, * *p* < 0.05, *** *p* < 0.001).

ESR analysis revealed significantly less polymerization free radical production from PMMA-TBB cements compared to PMMA-BPO cements, regardless of their age (Figure 8). Free radical levels were consistently low from one-day-old to seven-day-old PMMA-TBB specimens. By contrast, free radical levels were highest for one-day-old specimens, and maintained high even for seven-day-old specimens in PMMA-BPO cements. Of note, even in seven-day-old specimens, PMMA-TBB showed a substantially lower radical production than PMMA-BPO cements.

## 4. Discussion

This study addressed a clinically and scientifically important question about whether PMMA-TBB has an extended post-polymerization biological advantage over PMMA-BPO bone cements. Cell culture experiments on biomaterials are usually conducted by inoculating cells onto freshly prepared materials of interest to establish the immediate behavior and reaction of cells after first exposure to the biomaterial [20,21,39,40]. However, biomaterials, in particular bone cement, are not removable after use and are expected to remain at the surgical site for as long as the prosthesis lasts. Cells will continue to migrate to the delivered bone cement, and there will always be new interactions between cells and bone cement while the bone cement ages. To determine the ongoing biological performance of bone cements over time and mimic their persistence at surgical sites, here we prepared one-day-, three-day-, and seven-day-old bone cement specimens to simulate the time elapsed since surgery.

Our results consistently and comprehensively showed that PMMA-TBB has prolonged and superior biocompatibility over the two PMMA-BPO bone cements tested here. PMMA-TBB had a lower level of cytotoxicity that lasted for at least seven days, as demonstrated by the significantly increased number of osteoblasts surviving, attaching, proliferating, and differentiating on the material. Differentiation was evaluated according to ALP activity and the expression of differentiation-associated genes, which produced consistent results. This enhanced differentiation then resulted in increased extracellular matrix calcium deposition on PMMA-TBB, which was again demonstrated using multiple assays (alizarin red staining, chemical detection of calcium, and elemental analysis).

Regardless of the type of bone cement used, there was a general trend towards reduced cytotoxicity as the material aged, as shown by the day seven cell densities and ALP activity and day 15 calcium deposition, confirming that cytotoxicity reduces as polymerization progresses. There was also significantly greater cell density, ALP activity, and calcium deposition on seven-day-old specimens compared to three-day-old PMMA-BPO cements, suggesting a notable reduction in cytotoxicity between days three and seven after preparation. By contrast, the greatest improvement in cell density on PMMA-TBB was between one-day-old and three-day-old specimens or even earlier, suggesting that the rapid reduction in cytotoxicity occurred between zero and three days for PMMA-TBB.

The early cell attachment experiments suggest that the cytotoxicity of PMMA-TBB is rapidly mitigated. The number of cells attaching increased steadily on older PMMA-TBB specimens, with a large increase between one-day-old and three-day-old specimens. This assay was conducted after two days of culture, also confirming a rapid reduction in cytotoxicity. By contrast, the number of attached cells did not increase on older PMMA-BPO specimens, indicating persistent cytotoxicity. In fact, the radical level generated in seven-day-old specimens did not show a significant reduction compared to that in one-day-old specimens for PMMA-BPO cements tested. However, the reason why the number of cells attached at day 2 of culture decreased from one-day- to seven-day-old PMMA-BPO bone cements is unknown, and further studies to conduct other chemical characterizations are required, such as the amount and property change of residual monomers and other ingredients, such as N,N-dimethyl-p-toluidine (DmpT). Taken together, PMMA-TBB exhibited not only a higher baseline biocompatibility, but also an increase in biocompatibility over time compared to PMMA-BPO bone cements.

The WST-1 assay might not precisely enumerate cells [41,42]. There was an over 100-fold difference in WST-1 assay results between PMMA-BPO and PMMA-TBB materials depending on the age of the specimens, implying that an overwhelmingly greater number of cells survived and attached to PMMA-TBB. Albeit qualitative, the fluorescent microscopy observation of the cultures showed a 10–20-fold difference, which may be more representative of the actual difference between the two materials. The WST-1 assay is commonly used to quantify cell number and the assay measures the amount of a bio-reduced agent, formazan, as a result of the glycolytic production of (nicotinamide adenine dinucleotide phosphate) NADPH in viable cells [41,42,43]. Therefore, the assay quantifies cell number assuming that the cellular metabolic activity is equal between experimental groups. However, as seen from our cellular behavior and cytomorphometry results, cells grown on PMMA-TBB appeared healthier and conceivably had a higher metabolic activity, which might explain why there was a discrepancy between the WST-1 assay result and the microscopic observation. Nonetheless, it is worth mentioning that the metabolic activity of osteoblasts, which was significantly impaired on PMMA-BPO bone cements, was significantly restored on PMMA-TBB.

There was a difference in the expression trait between the early-stage and late-stage osteoblastic genes. Collagen 1, an early maker of osteoblastic differentiation, was only upregulated in response to PMMA-TBB during the early culture stages, whereas osteocalcin, a late-stage maker, was upregulated until the later stages of culture. Therefore, PMMA-TBB not only promoted but also maintained the expression of late-stage genes, as supported by the consistent upregulation of *Runx2* and *Osterix*, two osteoblastic transcription factors necessary for osteoblastic differentiation [44,45].

This study also assessed the polymerizing behavior of the materials using an established ESR to measure free radical production [20,30], with the results firmly supporting other cell culture experiments. It was surprising that PMMA-BPO maintained some cytotoxicity even on seven-day-old specimens, considering that the culture medium was replaced every three days. Cellular function, including attachment, proliferation, and differentiation, was severely compromised on the specimens, suggesting the continuous production of polymerization radicals from the materials even seven days after preparation. PMMA-BPO bone cements produced 5–10-fold more free radicals than PMMA-TBB on day seven post-preparation. Continuing radical production over 14 days within PMMA-BPO cements has been reported in other studies [19,20]. This explains why toxicity remains even after changing culture media every three days on PMMA-BPO cements. Hypothetical chemical structures have been proposed for PMMA-BPO and PMMA-TBB cements, but the exact mechanism during polymerization is unknown for both materials [19,20,30,46]. Further studies are required to identify the mechanism behind the remarkably reduced production of radicals in PMMA-TBB.

There were limitations to the present study. We chose the two most commonly used bone cement products as representative PMMA-BPO bone cement materials. Although the formulation of major components is the same among different products, the concentration of DmpT and the ingredients of polymers vary among the products, which may affect their biocompatibility. Comparison of the presently tested PMMA-TBB cement with other PMMA-BPO products will be necessary. To prevent confusion and complexity of data, and also due to technical difficulty, we chose seven-day-old bone cement for selected experiments such as cytomorphometry, SEM examination, and real-time PCR assay, instead of conducting these with all of the aging groups. It is known that bone cement materials reduce their toxicity as polymerization progresses. In other words, aged bone cements are assumed to show a less inter-product variation in biocompatibility. Therefore, we postulated that if we used older samples and detected the differences among three different bone cement types, it would be most reliable to conceivably represent the results of other aging groups. Although this study has revealed significance differences between the three cement types even after polymerization, it is apparent that this study is exploratory and needs a longer follow-up to fully address the question raised in the introduction. However, the present results were very successful and meaningful, providing assurance for further studies. Although this study presented interesting data with a focus on biological characterization, other chemical and mechanical characterizations will be of importance to comprehensively assess PMMA-TBB materials as a potential alternative to current PMMA-BPO cements.

Bone cement is usually considered bioinert and is encapsuled by inflammatory, fibrous tissue/cells or even necrotic tissue/cells once delivered to bone, as a consequence of its toxicity [20,47,48,49,50,51,52]. Further in vivo studies are warranted to examine how existing bone reacts to PMMA-TBB, and whether new bone formation occurs around PMMA-TBB. This study focused on a relatively short period of aging of bone cement. Future studies should have a longer-term follow-up to determine whether the superior biocompatibility of PMMA-TBB over PMMA-BPO persists for weeks, months, or indefinitely.

## 5. Conclusions

Here, we cultured osteoblasts on two commercially-available PMMA-BPO bone cements and an experimental PMMA-TBB cement aged for one, three, and seven days after polymerization. The number of surviving and attaching cells, the proliferative activity, and the rate of osteoblastic differentiation and mineralization were consistently and considerably higher on PMMA-TBB than on both PMMA-BPO cements, regardless of the age of the cement. Although osteoblasts were more functional on older specimens for all cement types, there were large performance improvements between three-day-old and seven-day-old specimens for PMMA-BPO cements, and between one-day-old and three-day-old specimens for PMMA-TBB. Free radical production was substantially higher from PMMA-BPO materials than PMMA-TBB, regardless of the age of the cement. These data suggest that PMMA-TBB has superior biocompatibility compared to PMMA-BPO materials over prolonged periods of at least seven days post-polymerization. This superior biocompatibility consisted of not only low baseline cytotoxicity, but also its further rapid reduction, suggesting a new, clinically relevant biological advantage for PMMA-TBB cement.

## Figures and Tables

**Table 1:** The constituents of three different bone cement materials used in this study.

Bone Cement Materials	Constituents
PMMA-BPO1 (Surgical Simplex P, Stryker, Kalamazoo, MI)	Powder: Polymethyl methacrylate (PMMA) (15.00%) Methyl methacrylate-styrene copolymer (73.70%) Benzoyl peroxide (BPO) initiator (1.30%) Barium sulfate (10.00%) Liquid: Methyl methacrylate (MMA) (97.40%) N,N-dimethyl p-toluidine (DmpT) (2.60%) Hydroquinone (HQ) (75 ppm)
PMMA-BPO2 (Endurance MV, DePuy Synthes, Warsaw, IN)	Powder: Polymethyl methacrylate (PMMA) (67.05%) Methyl methacrylate/styrene copolymer (21.10%) Benzoyl peroxide (BPO) initiator (1.85%) Barium sulfate (10.00%) Liquid: Methyl methacrylate (MMA) (98.00%) N,N-dimethyl-p-toluidine (DmpT)(< 2.00%) Hydroquinone (HQ) (75 ppm)
PMMA-TBB (Experimental)	Powder: Polymethyl methacrylate (PMMA) (90.00%) Barium sulfate (10.00%) Liquid: Methyl methacrylate (MMA) (91.00%) tri-n-butyl borane (TBB) initiator (9.00%) Hydroquinone (HQ) (50 ppm)

## Data Availability

The data presented in this study are available on request from the cooresponding author.

## References

[1] Mertz L. (2017). The Coming Gray Tide: Wanted: Health Innovations for an Increasingly Older Population. IEEE Pulse.

[2] Bonafede M., Shi N., Barron R., Li X., Crittenden D., Chandler D. (2016). Predicting imminent risk for fracture in patients aged 50 or older with osteoporosis using US claims data. Arch. Osteoporos..

[3] Li X.-Z., Zhang S.-N. (2020). Recent advance in treatment of osteoarthritis by bioactive components from herbal medicine. Chin. Med..

[4] Akkoc N., Khan M.A. (2020). Is Axial Spondyloarthritis More Common Than Rheumatoid Arthritis?. Curr. Rheumatol. Rep..

[5] Jakubaszek M., Płaza M., Kwiatkowska B. (2020). Color fraction as a useful method of imaging synovium vascularization in patients with high activity of rheumatoid arthritis. Reumatologia.

[6] Kremers H.M., Larson D.R., Crowson C.S., Kremers W.K., Washington R.E., Steiner C.A., Jiranek W.A., Berry D.J. (2015). Prevalence of Total Hip and Knee Replacement in the United States. J. Bone Jt. Surg. Am. Vol..

[7] Kurtz S., Mowat F., Ong K., Chan N., Lau E., Halpern M. (2005). Prevalence of Primary and Revision Total Hip and Knee Arthroplasty in the United States From 1990 Through 2002. J. Bone Jt. Surg. Am. Vol..

[8] Kurtz S., Ong K., Lau E., Mowat F., Halpern M. (2007). Projections of Primary and Revision Hip and Knee Arthroplasty in the United States from 2005 to 2030. J. Bone Jt. Surg. Am. Vol..

[9] Day J.S., Lau E., Ong K.L., Williams G.R., Ramsey M.L., Kurtz S.M. (2010). Prevalence and projections of total shoulder and elbow arthroplasty in the United States to 2015. J. Shoulder Elb. Surg..

[10] Bakhtiari S.S.E., Bakhsheshi-Rad H.R., Karbasi S., Tavakoli M., Razzaghi M., Ismail A.F., Ramakrishna S., Berto F. (2020). Polymethyl Methacrylate-Based Bone Cements Containing Carbon Nanotubes and Graphene Oxide: An Overview of Physical, Mechanical, and Biological Properties. Polymers.

[11] Melo-Fonseca F., Miranda G., Domingues H.S., Pinto I.M., Gasik M., Silva F.S. (2020). Reengineering Bone-Implant Interfaces for Improved Mechanotransduction and Clinical Outcomes. Stem Cell Rev. Rep..

[12] Bistolfi A., Ferracini R., Albanese C., Vernè E., Miola M. (2019). PMMA-Based Bone Cements and the Problem of Joint Arthroplasty Infections: Status and New Perspectives. Materials.

[13] Randall D.J., Anderson M.B., Gililland J.M., Peters C.L., Pelt C.E. (2019). A potential need for surgeon consensus: Cementation techniques for total knee arthroplasty in orthopedic implant manufacturers’ guidelines lack consistency. J. Orthop. Surg..

[14] Pacheco K.A. (2019). Allergy to Surgical Implants. Clin. Rev. Allergy Immunol..

[15] Duffau P., Beylot-Barry M., Palussière J., Ly S., Cogrel O., Doutre M.-S. (2006). Necrotic livedo after vertebroplasty. Br. J. Dermatol..

[16] Abu-Amer W., Arra M., Clohisy J.C., Abu-Amer Y., Swarnkar G. (2019). Targeting vascular endothelial growth factor ameliorates PMMA-particles induced inflammatory osteolysis in murine calvaria. Bone.

[17] Singh V., Bhakta P., Zietak E., Hussain A., Zietak E. (2016). Bone cement implantation syndrome: A delayed postoperative presentation. J. Clin. Anesthesia.

[18] Paz E., Ballesteros Y., Abenojar J., Del Real J., Dunne N. (2019). Graphene Oxide and Graphene Reinforced PMMA Bone Cements: Evaluation of Thermal Properties and Biocompatibility. Materials.

[19] Nakagawa K., Saita M., Ikeda T., Hirota M., Park W., Lee M.C.-I., Ogawa T. (2015). Biocompatibility of 4-META/MMA-TBB resin used as a dental luting agent. J. Prosthet. Dent..

[20] Tsukimura N., Yamada M., Aita H., Hori N., Yoshino F., Lee M.C.-I., Kimoto K., Jewett A., Ogawa T. (2009). N-acetyl cysteine (NAC)-mediated detoxification and functionalization of poly(methyl methacrylate) bone cement. Biomaterials.

[21] Yamada M., Kojima N., Paranjpe A., Att W., Aita H., Jewett A., Ogawa T. (2008). N-acetyl Cysteine (NAC)-assisted Detoxification of PMMA Resin. J. Dent. Res..

[22] Ciapetti G., Granchi D., Cenni E., Savarino L., Cavedagna D., Pizzoferrato A. (2000). Cytotoxic effect of bone cements in HL-60 cells: Distinction between apoptosis and necrosis. J. Biomed. Mater. Res..

[23] Yamada M., Ogawa T. (2009). Chemodynamics underlying N-acetyl cysteine-mediated bone cement monomer detoxification. Acta Biomater..

[24] Aita H., Tsukimura N., Yamada M., Hori N., Kubo K., Sato N., Maeda H., Kimoto K., Ogawa T. (2010). N-acetyl cysteine prevents polymethyl methacrylate bone cement extract-induced cell death and functional suppression of rat primary osteoblasts. J. Biomed. Mater. Res. Part A.

[25] Goiato M.C., Freitas E., Dos Santos D., De Medeiros R., Sonego M. (2015). Acrylic Resin Cytotoxicity for Denture Base: Literature Review. Adv. Clin. Exp. Med..

[26] Oonishi H., Ohashi H., Kawahara I. (2016). Total Hip Arthroplasty around the Inception of the Interface Bioactive Bone Cement Technique. Clin. Orthop. Surg..

[27] Blom E.J., Klein-Nulend J., Klein C.P., Kurashina K., van Waas M.A., Burger E.H. (2000). Transforming growth factor-beta1 incorporated during setting in calcium phosphate cement stimulates bone cell differentiation in vitro. J. Biomed. Mater. Res..

[28] Elisabettacenni E., Granchi D., Ciapetti G., Savarino L., Vancini M., Di Leo A. (2001). Effect of CMW 1 bone cement on transforming growth factor-beta 1 expression by endothelial cells. J. Biomater. Sci. Polym. Ed..

[29] Bigi A., Torricelli P., Fini M., Bracci B., Panzavolta S., Sturba L., Giardino R. (2004). A Biomimetic Gelatin-Calcium Phosphate Bone Cement. Int. J. Artif. Organs.

[30] Hamajima K., Ozawa R., Saruta J., Saita M., Kitajima H., Taleghani S.R., Usami D., Goharian D., Uno M., Miyazawa K. (2020). The Effect of TBB, as an Initiator, on the Biological Compatibility of PMMA/MMA Bone Cement. Int. J. Mol. Sci..

[31] Sugita Y., Saruta J., Taniyama T., Kitajima H., Hirota M., Ikeda T., Ogawa T. (2020). UV-Pre-Treated and Protein-Adsorbed Titanium Implants Exhibit Enhanced Osteoconductivity. Int. J. Mol. Sci..

[32] Saruta J., Sato N., Ishijima M., Okubo T., Hirota M., Ogawa T. (2019). Disproportionate Effect of Sub-Micron Topography on Osteoconductive Capability of Titanium. Int. J. Mol. Sci..

[33] Hasegawa M., Saruta J., Hirota M., Taniyama T., Sugita Y., Kubo K., Ishijima M., Ikeda T., Maeda H., Ogawa T. (2020). A Newly Created Meso-, Micro-, and Nano-Scale Rough Titanium Surface Promotes Bone-Implant Integration. Int. J. Mol. Sci..

[34] Ghassemi A., Ishijima M., Hasegawa M., Rezaei N.M., Nakhaei K., Sekiya T., Torii Y., Hirota M., Park W., Miley D.D. (2018). Biological and Physicochemical Characteristics of 2 Different Hydrophilic Surfaces Created by Saline-Storage and Ultraviolet Treatment. Implant. Dent..

[35] Taniyama T., Saruta J., Rezaei N.M., Nakhaei K., Ghassemi A., Hirota M., Okubo T., Ikeda T., Sugita Y., Hasegawa M. (2020). UV-Photofunctionalization of Titanium Promotes Mechanical Anchorage in A Rat Osteoporosis Model. Int. J. Mol. Sci..

[36] Sakaguchi W., Kubota N., Shimizu T., Saruta J., Fuchida S., Kawata A., Yamamoto Y., Sugimoto M., Yakeishi M., Tsukinoki K. (2020). Existence of SARS-CoV-2 Entry Molecules in the Oral Cavity. Int. J. Mol. Sci..

[37] Shiotsu-Ogura Y., Yoshida A., Kan P., Sasaki H., Toyama T., Izukuri K., Hamada N., Yoshino F., Shiotusu-Ogura Y. (2019). Antimicrobial photodynamic therapy using a plaque disclosing solution on Streptococcus mutans. Photodiagnosis Photodyn. Ther..

[38] Ozawa R., Saita M., Sakaue S., Okada R., Sato T., Kawamata R., Sakurai T., Hamada N., Kimoto K., Nagasaki Y. (2020). Redox injectable gel protects osteoblastic function against oxidative stress and suppresses alveolar bone loss in a rat peri-implantitis model. Acta Biomater..

[39] Yamada M., Kojima N., Att W., Hori N., Suzuki T., Ogawa T. (2009). N-Acetyl cysteine restores viability and function of rat odontoblast-like cells impaired by polymethylmethacrylate dental resin extract. Redox Rep..

[40] Kojima N., Yamada M., Paranjpe A., Tsukimura N., Kubo K., Jewett A., Ogawa T. (2008). Restored viability and function of dental pulp cells on poly-methylmethacrylate (PMMA)-based dental resin supplemented with N-acetyl cysteine (NAC). Dent. Mater..

[41] Yin L.-M., Wei Y., Wang W.-Q., Wang Y., Xu Y.-D., Yang Y.-Q. (2014). Simultaneous application of BrdU and WST-1 measurements for detection of the proliferation and viability of airway smooth muscle cells. Biol. Res..

[42] Joo K.-M., Kim S., Koo Y.J., Lee M., Lee S.-H., Choi D., Lim K.-M. (2019). Development and validation of UPLC method for WST-1 cell viability assay and its application to MCTT HCE™ eye irritation test for colorful substances. Toxicol. Vitr..

[43] Lutter A.-H., Scholka J., Richter H., Anderer U. (2017). Applying XTT, WST-1, and WST-8 to human chondrocytes: A comparison of membrane-impermeable tetrazolium salts in 2D and 3D cultures. Clin. Hemorheol. Microcirc..

[44] Lian J.B., Javed A., Zaidi S.K., Lengner C., Montecino M., Van Wijnen A.J., Stein J.L., Stein G.S. (2004). Regulatory controls for osteoblast growth and differentiation: Role of Runx/Cbfa/AML factors. Crit. Rev. Eukaryot. Gene Expr..

[45] Nakashima K., Zhou X., Kunkel G., Zhang Z., Deng J.M., Behringer R.R., de Crombrugghe B. (2002). The Novel Zinc Finger-Containing Transcription Factor Osterix Is Required for Osteoblast Differentiation and Bone Formation. Cell.

[46] Sugita Y., Okubo T., Saita M., Ishijima M., Torii Y., Tanaka M., Iwasaki C., Sekiya T., Tabuchi M., Rezaei N.M. (2020). Novel Osteogenic Behaviors around Hydrophilic and Radical-Free 4-META/MMA-TBB: Implications of an Osseointegrating Bone Cement. Int. J. Mol. Sci..

[47] Revell P., Braden M., Freeman M. (1998). Review of the biological response to a novel bone cement containing poly(ethyl methacrylate) and n-butyl methacrylate. Biomaterials.

[48] Johnston R.C. (2005). Acrylic bone cement: Clinical development and current status in North America. Orthop. Clin. N. Am..

[49] Granchi N., Cenni E., Savarino L., Ciapetti G., Forbicini G., Vancini M., Maini C., Baldini N., Giunti A. (2002). Bone cement extracts modulate the osteoprotegerin/osteoprotegerin-ligand expression in MG63 osteoblast-like cells. Biomaterials.

[50] Cenni E., Granchi D., Ciapetti G., Savarino L., Corradini A., Vancini M., Giunti A. (2002). Gene expression of bone-associated cytokines in MG63 osteoblast-like cells incubated with acrylic bone cement extracts in minimum essential medium. J. Biomater. Sci. Polym. Ed..

[51] Granchi D., Ciapetti G., Filippini F., Stea S., Elisabettacenni E., Pizzoferrato A., Toni A. (2000). Modulation of pro- and anti-apoptotic genes in lymphocytes exposed to bone cements. J. Biomater. Sci. Polym. Ed..

[52] Tsukeoka T., Suzuki M., Ohtsuki C., Sugino A., Tsuneizumi Y., Miyagi J., Kuramoto K., Moriya H. (2006). Mechanical and histological evaluation of a PMMA-based bone cement modified with gamma-methacryloxypropyltrimethoxysilane and calcium acetate. Biomaterials.

